# Coverage and usage of insecticide treated nets (ITNs) within households: associated factors and effect on the prevalance of malaria parasitemia in the Mount Cameroon area

**DOI:** 10.1186/s12889-019-7555-x

**Published:** 2019-09-03

**Authors:** Charles Njumkeng, Tobias O. Apinjoh, Judith K. Anchang-Kimbi, Elvis T. Amin, Elvis A. Tanue, Clarisse Njua-Yafi, Eric A. Achidi

**Affiliations:** 1 Global Research Education and health Foundation Buea, Molyko, P.O. BOX 356, Buea, South West Region Cameroon; 2 Department of Biochemistry and Molecular Biology, University of Buea, Buea, Cameroon; 3 Department of Microbiology and Parasitology, University of Buea, Buea, Cameroon; 4 Department of Zoology and Animal Physiology, University of Buea, Buea, Cameroon; 5 Department of Public Health and Hygiene, University of Buea, Buea, Cameroon; 6 Department of Animal Biology and Physiology, University of Yaounde I, Yaounde, Cameroon; 7 College of Technology, University of Bamenda, Bamenda, Cameroon

**Keywords:** Coverage, Usage, ITNS, Households, Malaria, Cameroon

## Abstract

**Background:**

Insecticide-treated nets (ITNs) are a widely used tool that has been proven to be effective in the prevention and control of malaria in malaria endemic countries. However, usage varies among households and can greatly affect the benefits of ITNs as a control tool for malaria transmission. This study determined the coverage and usage of ITNS as well as associated factors and the effect of coverage and usage on the prevalence of malaria parasitemia within households in the Mount Cameroon area.

**Methods:**

A cross-sectional survey was conducted between August and September 2014 in six communities within the Mount Cameroon area. Households within the communities were enrolled through multistage sampling and household survey was done using a structured questionnaire. Capillary blood was collected for malaria parasite determination. Data was analysed using SPSS version 20 for windows. Differences in proportions were assessed using the Chi-square test while factors affecting ITNs usage were assessed in multivariate logistic regression at a statistical significance of *P* ≤ 0.05.

**Results:**

A total of 504 households were surveyed, 1564 bed spaces reported while 915(58.5, 95% CI: 56.1–60.9) of the bed spaces had nets and 391(77.6, 95% CI, 74.0–80.2) of the households had at least one bed net. The odds of using ITNs was 2 folds higher (OR = 2.41; 95% CI 1.58–3.69 *p* = 0.001) and 3 folds higher (OR = 3.149, 95% CI 1.53–6.47 *p* = 0.002) among houses with 5 to 9 occupants and above 10 occupants respectively when compared to houses with less than 5 occupants. In addition, Individuals living in cement block houses were less likely to use ITNs.

Compared to those living in wooden houses (OR = 0.488, 95% CI: 0.269–0.885; p = 0 .018). Rural communities had lower ITN coverage compared to semi-urban communities (*p* = 0.0001). Increase in ITNs coverage significantly reduces malaria prevalence (correlation − 0.899, *p* = 0.015).

**Conclusion:**

Despite the efforts made to scale up ITN distribution so that universal coverage can be attained, coverage remains low. Increasing coverage and putting in place a mechanism to replace torn nets will go a long way reduce the prevalence of malaria parasitemia.

**Electronic supplementary material:**

The online version of this article (10.1186/s12889-019-7555-x) contains supplementary material, which is available to authorized users.

## Background

Malaria is one of the major public health problems with estimated 212 million malaria cases and 429, 000 malaria related death across the globe in 2016. In Africa, access to the tools needed for the prevention and treatment of malaria is still limited to millions of people in need [1]. In Cameroon, the Ministry of Public Health through the National Malaria Control Program (NMCP) has put in place a number of strategies to fight this disease. This includes insecticide treated nets (ITNS), free treatment of uncomplicated malaria for children below 5 years of age using Artemisinin-based combination therapies (ACTs), and prompt management of confirmed cases with Artemisinin-based Combination Therapy, intermittent preventive treatment with sulphadoxine-pyrimethamine for pregnant women, environmental hygiene and in-door residual spraying [2]. From 2002 the Cameroon government has conducted many campaigns for the free distribution of ordinary ITNs and long lasting insecticides treated ITNs all over the country [3]. According to the Ministry of Public Health about two million ITNs were distributed between 2005 and 2008, over eight million distributed in 2011 and about twelve million in 2015 as a strategy to significantly reduce the morbidity and mortality burden of malaria in the country [6].

Despite the government’s effort to scale up ITN distribution in Cameroon, full coverage and proper usage may be limited due to the lack of consistent distribution and other issues related to maintenance and replacement of nets, as well as local beliefs and poor understanding of the relationship between mosquitoes and malaria at the community level [4–6]. In addition, studies have observed great disparities between ownership versus usage of ITNs [3, 7–11].

To acquire the anticipated benefits of ITNs, they should be properly and consistently used within households rather than merely owning them. WHO recommends that periodic household surveys should be done to determine whether the populations at risk have sufficient ITNs and whether they are using them properly [12]. Community and household factors such as discomfort, belief of low mosquito density, inconvenience installing the nets, residence, educational background, age and gender differences are among the frequently reported reasons for not using the acquired ITNs [3, 6, 13, 14].

In a previous publication [5] it was demonstrated that ITN usage reduces the prevalence of malaria parasitemia which is considered as a measure of malaria burden. However, this publication did not include an analysis of community and household specific factors affecting coverage and proper usage of ITNs. This study used an unbiased approach to analyse and identify factors affecting ITN usage at household and community levels so that they can be addressed to reduce malaria transmission.

## Methods

### Ethical considerations

Ethical approval for the study was obtained from the Institutional Review Board of the Faculty of Health Sciences, University of Buea (N^0^: 2014-02-0191). Administrative authorization was obtained from the South West Regional Delegation of Public Health and from the District Medical Officer of Tiko and Buea. Written informed consent was obtained from participants after the purpose of the study and the role of the participants was well explained verbally and in the consent form.

### Study area

The study was carried out in six major settlements (Ombe, Mutengene, Dibanda, Bolifamba, Muea and Tole) selected along the eastern slope of Mt Cameroon with different malaria transmission profiles and geographic features [3, 15]. Ombe and Tole are rural communities while Mutengene, Dibanda, Bolifamba and Muea are semi-urban communities. The communities included in this study are characterized by small bushes and stagnant water (during the raining seasons) that favors anopheles breeding throughout the year [16]. The population of this area is comprised of many ethnic groups, majority coming from neighboring regions in search of its fertile volcanic land for farming and business opportunities. This area has two main house types, namely the cement brick and wooden houses [5, 16–18].

The area has moved from a Malaria hyper endemic area to a meso-endemic area, with maximum transmission occurring during the rainy seasons and at lower altitude [19, 20]. Majority of the malaria infections in this area are cause by the *P. falciparum* while *Anopheles gambiae* has been identified as the dominant and most important malaria vector in the area [5, 19, 21] *A. gambiae* annual biting rate is estimated as 287 infective bites per person while daily the overall Entomological Inoculation Rates (EIR) is estimated to be 3.93 infective bites per person [5, 22].

### Study design and sampling technique

This study was a cross-sectional household survey which was conducted between August and September 2014. The communities were randomly selected along eastern the slop of Mount Cameroon based on altitude which is an indicator of malaria transmission [5]. Within each selected community, a household was selected at random as a starting point and the rest selected after every five households (Systematic sampling technique). The number of households to skip before enrolling one (five households) was calculated by dividing the total number of households in the community by the number of households required for the study.

Structured questionnaires (Additional file 1) were then administered to heads of households (with priority given to mothers) and when faced with a situation where the head of the household was absent, any adult within the household able of providing reliable information was interviewed. Pidgin English was used to administer the questionnaires. All reported ITNs were inspected except in some cases where inspection was not possible (one of the rooms is locked and the key is not readily available). Community health workers were trained to administer the questionnaires and they were supervised during the exercise. Before the survey, the questionnaire was tested in a non-survey population to ensure validity of the pre-coded answers. Capillary blood samples were collected from individuals living within the selected communities for malaria parasite examination. The community members were sensitized prior to each sample collection visit.

### Malaria Parasitemia examination

After cleaning the lobe of a finger with cotton wool soaked in 70% ethanol, the finger was pricked using a sterile lancet. Two freefall drops of blood were collected from the finger pricked and used to prepare thick and thin blood films on labeled slides and then air dried. The thin films were fixed with methanol and both films were stained with 10% Giemsa (Sigma, St. Lothanoluis, USA) for 15 min. The malaria parasitaemia status and density were determined under oil immersion with the 100x objective, of a binocular Olympus microscope (Olympus Optical Co., Ltd., Japan) while the *Plasmodium* species were identified on the thin blood smear. A smear was only considered negative if no malaria parasites were seen in 50 high power fields [23].

### Sample size determination

The sample size of the study population was calculated using the formula below [24]:

$$ \mathrm{n}\kern1.5em =\kern1em \frac{\kern0.5em \left\{\frac{\mathrm{P}\left(1-\mathrm{P}\right)}{\frac{{\mathrm{A}}^2}{Z^2}+\frac{\mathrm{P}\left(1\hbox{-} \mathrm{P}\right)}{N}}\right\}}{\mathrm{R}} $$

Where: *n* = sample size required,

N = number of household in the community = 14,897 (sum of households in Mutengene, Ombe Bolifamba Muea and Tole),

P = estimated variance in population, as a decimal: (0.3 for 70–30), A = Precision desired, expressed as a decimal (i.e.0.05 for 5%), Z is based on confidence level: 1.96 for 95% confidence and R = Estimated net coverage, as a decimal (59.7%) [3].

A minimum of 451 households were required for the study. The number of households selected per community was calculated as a proportion of the sum of all the households.

### Definition of terms

A household was considered to be a home with a head, his / her dependents or spouse and who feed from the same kitchen and sleep under the same roof. Any long lasting insecticide treated net or a conventional ITN which was treated not more than 3 years ago was consider as ITN [5]. “Bed space” was defined as any space or any facility that is regularly used for sleeping or intended to be used for sleeping by someone.

ITN ownership was defined as being in possession of an insecticide treated bed net. The net could be in use or not in use but must be in a good state. Any net that had not been used even once since it was obtained, was termed net in package.

Regular users of ITNs were defined as those who slept under an ITN for at least the last two nights before the survey was conducted. An irregular user was defined as someone who had an ITN hung over his/her bed but reported that he/she did not sleep under the ITN for 1 or 2 days before the survey. Non users were defined as those who did not use ITNs at all. Any ITN that was found hung over a sleeping space was considered to be a net in use.

### Determination of ITN ownership and usage

The proportions of ITN ownership, usage and coverage were calculated respectively as follows:

$$ \mathrm{ITN}\mathrm{ownership}=\frac{\ \mathrm{Number}\ \mathrm{of}\ \mathrm{households}\ \mathrm{with}\ \mathrm{at}\ \mathrm{least}\ \mathrm{an}\ \mathrm{ITN}}{\mathrm{Total}\ \mathrm{number}\ \mathrm{of}\ \mathrm{households}\ \mathrm{surveyed}} $$

$$ \mathrm{ITNin}\ \mathrm{use}=\frac{\mathrm{Number}\ \mathrm{of}\ \mathrm{nets}\ \mathrm{found}\ \mathrm{hung}\ \mathrm{over}\ \mathrm{a}\ \mathrm{sleeping}\ \mathrm{space}\ }{\mathrm{Total}\ \mathrm{number}\ \mathrm{of}\ \mathrm{nets}\ \mathrm{reported}\ \mathrm{and}\ \mathrm{confirmed}} $$

$$ \mathrm{ITN}\ \mathrm{usage}\ \mathrm{rate}=\frac{\mathrm{Number}\ \mathrm{of}\ \mathrm{households}\ \mathrm{that}\ \mathrm{used}\ \mathrm{at}\ \mathrm{least}\ \mathrm{one}\ \mathrm{ITN}\ \mathrm{the}\ \mathrm{previous}\ \mathrm{night}\ }{\mathrm{Total}\ \mathrm{number}\ \mathrm{of}\ \mathrm{households}\ \mathrm{with}\ \mathrm{at}\ \mathrm{least}\ \mathrm{one}\ \mathrm{ITN}} $$

$$ \mathrm{ITNcoverage}=\frac{\mathrm{Total}\ \mathrm{number}\ \mathrm{of}\ \mathrm{ITNsreported}\ }{\mathrm{Total}\ \mathrm{number}\ \mathrm{of}\ \mathrm{sleeping}\ \mathrm{spaces}\ \mathrm{reported}} $$

### Data management and statistical analysis

Data obtained from each participant was entered into the research log books and checked by the lead author. The data were then entered into Epi info version 7.0 software. Any discrepancy in field records was verified using the original questionnaires. After data cleaning, analysis was done using SPSS Statistics 20.0 Statistical software for windows (SPSS Inc., Chicago USA). ITN coverage per household was expressed as means ± SD. The comparison between ITN coverage, ownership, usage and the nature of nets between different study sites was assessed using the Chi-square test. The association between net usage and factors affecting its use was done using bivariate logistic regression and adjusted using multivariate logistic regression. A bivariate logistic regression was also used to determine the relationship between ITN usage and the prevalence of malaria. Pearson correlation was used to determine the relationship between ITN coverage and the prevalence of malaria parasitemia. Statistical significance was designated at *p* ≤ 0.05.

## Results

A total of 504 households were surveyed amongst which 295 (58.5%) were headed by individuals who had acquired at most primary level education while 58 (11.5%) were individuals who had attained at least higher education. With respect to the various sites, participants from Ombe were dominated 43(82.7%) by those who attended at most primary education compared to Muea and Dibanda with a higher education attainment as their highest level of education 61(56.5%) and 38(50.0%) respectively (*p* < 0.0001).

Regarding the nature of the houses surveyed 54(88.5%) of the 61 houses found in Tole were wooden houses followed by Bolifamba with 45(63.4%) of 71 houses surveyed while majority of houses in Mutenegene 96(70.6) and Dibanda 49(64.5%) were cement block houses (*p* < 0.0001). No significant difference was found between number of occupants in a household and the various communities involved (p, 0.125).

A total of 915 ITNs were found within the households surveyed. During inspection it was discovered that 490 (53.6%) of the nets were torn and the highest number of torn nets was found in Dibanda 99(74.4%) of 133 net compared to 101(42.1%) of 140 nets found in Mutengene (*p* = 0.011). The characteristics of respondents are presented on Table 1.

**Table 1:** Characteristics of respondents in the Mount Cameroon area September, 2014

Factor	Category	Site N (%)							
		Total	Tole	Muea	Bolifamba	Dibanda	Mutenegene	Ombe	*P* value
Level of education of the head of household	At most Primary	295	38 (62.3)	61 (56.5)	43 (60.6)	38 (50.0)	72 (52.9)	43 (82.7)	0.001
	Secondary	151	20 (32.8)	37 (34.3)	23 (32.4)	23 (30.0)	39 (28.7)	9 (17.3)	
	Higher	58	3 (4.9)	10 (9.3)	5 (7.0)	15 (19.7)	25 (18.4)	0 (0.0)	
Nature of house	Cement block	235	7 (11.5)	45 (41.7)	26 (36.6)	49 (64.5)	96 (70.6)	12 (23.1)	<0.00001
	Wooden house	269	54 (88.5)	63 (58.3)	45 (63.4)	27 (35.5)	40 (29.4)	40 (76.9)	
Nature of net	Torn	490	24 (52.2)	147 (53.1)	76 (50.7)	99 (74.4)	101 (42.1)	43 (62.3)	0.011
	Good	425	22 (47.8)	130 (46.93)	74 (49.33)	34 (25.56)	139 (57.91)	26 (37.68)	
Number of households surveyed		504	61 (12.1)	108 (21.4)	71 (14.1)	76 (15.1)	136 (27.0)	52 (10.3)	–
Number of occupants in a household	1–4	167	21 (34.4)	29 (26.9	21 (29.6)	30 (39.5)	49 (36.3)	17 (32.7)	0.125
	5–9	272	38 (62.3)	58)53.7)	40 (56.3)	36 (47.4)	74 (54.8)	26 (50.0)	
	≥10	64	2 (3.3)	21 (19.4)	10 (14.1)	10 (13.2)	12 (8.9)	21 (19.4)	

### Ownership and usage of ITNs within households in the Mount Cameroon area

From Table 2 of the 504 households surveyed, 391 (77.6%) owned at least a net. Within the houses 1564 bed spaces were recorded and 915 ITNs were reported and confirmed giving a coverage of 58.5% of bed spaces and an average of 1.8 (SD 1.3) ITNs per household. Tole had the least coverage (25.7% coverage of bed spaces and 52.5% coverage of households) while Muea had the highest coverage (78.9% coverage of total bed spaces and 88.9% coverage of households) *p* < 0.0001. A total of 3040 persons were declared in the households with 2459 (80.89%) belonging to the households with at least one net.

**Table 2:** ITN coverage in the Mount Cameroon Area, September 2016

Study Site	Coverage			
	Number of households surveyed (H)	Number of bed spaces reported (N)	Number of ITNs found (N %)	Households with At least one ITNs (H %)
Ombe	52	174	69 (39.7)	38 (73.1)
Tole	61	179	46 (25.7)	32 (52.5)
Mutengene	136	406	240 (59.1)	115 (84.6)
Dibanda	76	246	133 (54.1)	43 (56.6)
Bolifamba	71	208	150 (72.1)	63 (88.7)
Muea	108	351	277 (78.9)	96 (88.9)
Overall	504	1564	915 (58.5)	387 (76.8)
*P*-value	**–**	**–**	<0.0001	<0.0001

As shown in Table 3, of the 504 households surveyed, 367(94.8%) had at least one ITN in use while about 88% of the ITNs in the communities were in use. Tole had the least number of ITNs in use (67.4%) and Muea significantly higher (97.5%) *p* < 0.0001. In Muea and Dibanda, all the households that owned ITNs had at least one in use. Out of 391 households with at least one ITN, 303 slept under their ITNs the night before the survey giving an overall usage rate of 77.5% while 208 had slept under their ITNs for at least the last two nights before survey was conducted thus, an overall rate of 53.7 regular users. Tole had the lowest overall rate of regular ITNs usage of 31.3% while Dibanda had the highest usage rate of 68.09% *p* < 0.0001.

**Table 3:** ITN usage in the Mount Cameroon Area, September 2016

Study Site	ITNs Usage					
	Number of households surveyed H	Households with At least one ITNs = n (H %)	Number of ITNs found (N)	Number of ITNs in use %)	Households with at least one ITNs in use (n %)	Households with Regular ITN Usage (n %)
Ombe	52	38 (73.1)	69	65 (94.2)	37 (97.4)	26 (68.4)
Tole	61	32 (52.5)	46	31 (67.4)	26 (81.3)	10 (31.3)
Mutengene	136	115 (84.6)	240	196 (81.7)	105 (91.3)	35 (30.4)
Dibanda	76	43 (56.6)	133	129 (96.9)	43 (100)	32 (681)
Bolifamba	71	63 (88.7)	150	117 (78.0)	56 (88.9)	35 (55.5)
Muea	108	96 (88.9)	277	270 (97.5)	96 (100)	70 (72.9)
Overall	504	387 (76.8)	915	808 (88.3)	367 (94.8)	208 (53.7)
*P*-value		<0.0001	**–**	<0.0001	<0.0001	<0.0001

### ITNs that are not in use in the community

**Fig. 1:**
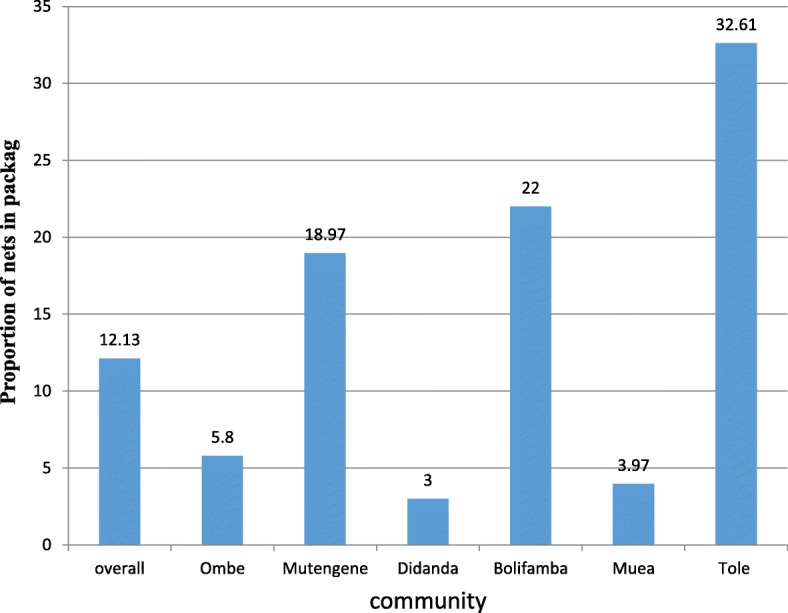
Proportion of nets that are not in use in the community

Out of 915 ITNs found in the communities 111(12.13%) were found folded up or in package and 11(1.2%) were used for other purposes such as window nets, to partition a room and on toilet doors. Tole had the highest number of ITNs in package (32.61%) followed by Bolifamba (22.0%) while Didanda had the least number of ITNs in package (3.0%) as shown in Fig. 1.

### Reason for not sleeping under the ITNthe day before the survey was conducted

**Fig. 2:**
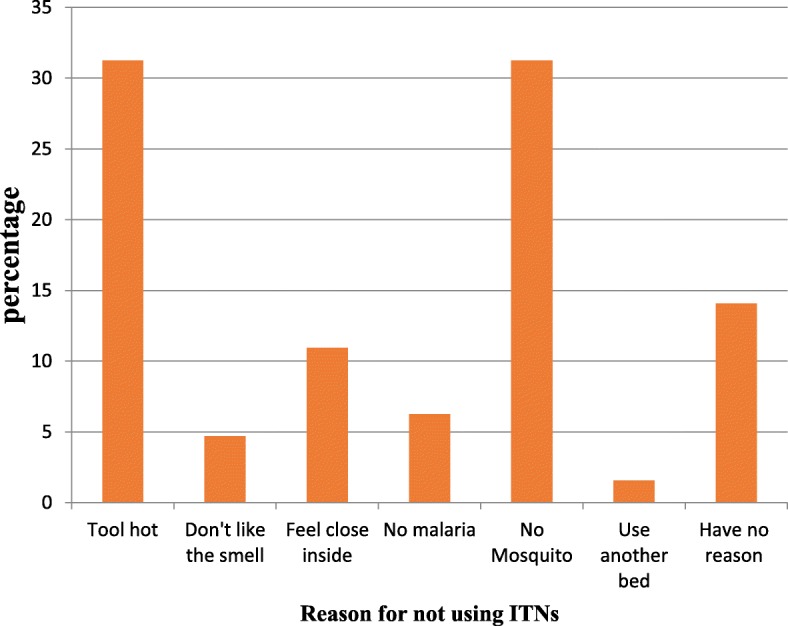
Reasons for not using ITNs within the households

**Fig. 3:**
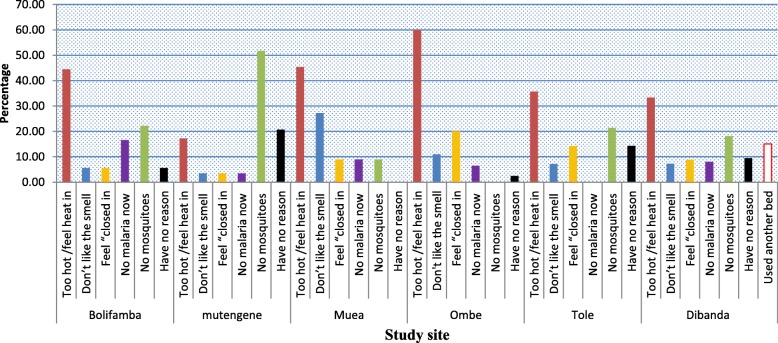
Community specific reasons for not using ITNs within the households

Several reasons were put forth by those who still had ITNs in package and those who did not sleep under their ITNs the previous night before the survey. In general the most frequently reported reasons for not sleeping under the net were “too hot/feel heat in” (33.25%) and the perception of “no mosquitoes” (33.25%) as shown on (Fig. 2). With respect to the various study sites, the reasons of not using ITNs was dominated by too hot/feel heat in. It is worth noting that the highest number of people who failed to use their net because of “too hot/feel heat in” was recorded in Ombe (60%). However in Mutengene the most common reason for not sleeping under net was the perception of no mosquitoes (50%) (Fig. 3).

### Bivariate logistic regression analysis of the association between net usage and possible explanatory factors

With respect to ITN usage at the level of the households it was observed that most of the households (87.6%) that had torn ITNs used them the night before the survey compared to households that had good ITNs (OR = 2.11, 95% CI: (1.16–3.83), *p* < 0.00001). It was also observed that ITN usage within households was higher among households with 5 to 9 occupants (OR = 2.41, 95% CI: 1.57–3.69, *p* = 0.0001) and those with 10 or more occupants (OR = 3.15, 95% CI: 1.53–6.47 *p* = 0.002) when compared to households with less than 5 occupants. Furthermore, ITNs usage was lower among individuals living in cement block houses (OR .488, 95%CI: 0.23–0.89; *p* = 0 .018) compared to those living in wooden houses as shown on Table 4.

**Table 4:** Bivariate logistic regression analysis of the association between net usage and possible explanatory factors

Factor	Category	Number of participants (n)	Used ITN (n%)	OR(95% CI)	P-value
Number of house occupants	< 5	167	101 (60.5)	1	
	5–9	272	214 (78.7)	2.41 (1.57–3.69)	0.001
	≥10	64	53 (82.8)	3.15 (1.53–6.47)	0.002
Level of education	At least Higher	58	17 (29.3)	1	
	Secondary	151	42 (27.8)	1.070 (0.55–2.10)	0.830
	At most Primary	295	76 (56.3)	1.11 (0.64–2.23)	0.575
Nature of house	Wooden	195	177 (90.8)	1	1
	Cement block	308	234 (76.2)	.49 (0.25–.89)	0.018
Nature of nlet	Torn	275	241 (87.6)	2.11 (1.16–3.83)	0.014
	Good	96	74 (77.1)	1	
Altitude	Low	188	61 (32.4)	0.607 (0.44–0.85)	0.003
	Intermediate	315	147 (46.7)	1.	
Community status	Rural	63()	36 (57.1)	.923 (0.44–1.95)	0.834
	Urban	304	213 (70.0)	1.0	
Gender	Male	1331	891 (66.9)	0.98 (0.73–1.32)	0.885
	Female	1708	1134 (66.4)	1	
Age Group (Years)	< 5	662	289 (43.7)	1.038 (0.72–1.50)	0.841
	5–9	626	254 (40.6)	1.178 (0.81–1.71)	0.393
	10–15	407	125 (30.8)	1.805 (1.14–2.86	0.012
	> 15	1340	598 (44.6)	1	1

1 is the reference group

With respect to altitude it was observed that ITNs usage is reduced at low altitude when compared to intermediated altitude (OR = 0.607, 95% CI: 0 .44–0.85, *p* = .003) while individual factors such as age group, level of education, and gender were not associated to ITN usage.

### Multivariate logistic regression analysis of the association between net usage and possible explanatory factors

The Multivariate model was done with five variables that were significantly associated with ITN usage in the bivariate analysis. This revealed that the most determinant factors associated with increased ITN usage were the nature of house and altitude. The odds of ITNs usage was reduced by 38% among households at low altitude compared to those at intermediate altitude (OR = 0.613, 95% CI: 0.44–0.86, *p* = .004). The nature of the house was also significantly associated with reduced ITN usage, likewise the odds for using ITNs was reduced by 51% among individuals living in cement block houses when compared to those living in wooden houses (OR = 0.49, 95% CI: 0.269–0.885, *P* = .018). After adjusting for other factors it was also observed that the odd of using ITNs was 87% higher among individuals with torn nets compared to those with good ITNs, (OR = 1.87, 95% CI: 1.133.10, *P* = .015) as presented on Table 5.

**Table 5:** Multivariate logistic regression analysis of the association between net usage and possible explanatory factors

Factor	Category	OR	95% CI	P-value	*P*-value *p*-value test for trend
Number of house occupants	<5	1			0.072
	5–9	0.99	(.32–3.06)	.989	
	≥10	0.48	(.18–1.29)	.149	
Nature of house	Wooden	1			0.016
	Cement block	0*.*49	0.269–0.885	***.***018	
Nature of net	Torn	1.87	1.13–3.10	0.015	0.00001
	Good	1			
Altitude	Low	.613	.44–.86	.004	0.003
	Intermediate	1.0			
Age Group (Years)	< 5	1.01	.70–1.46	.948	0.08
	5–9	1.145	.78–1.67	.489	
	10–15	1.75	1.10–2.78	.018	
	> 15	1.0			

1.0 is the reference group

### Relationship between ITN coverage, usage and malaria parasitemia prevalence

**Fig. 4:**
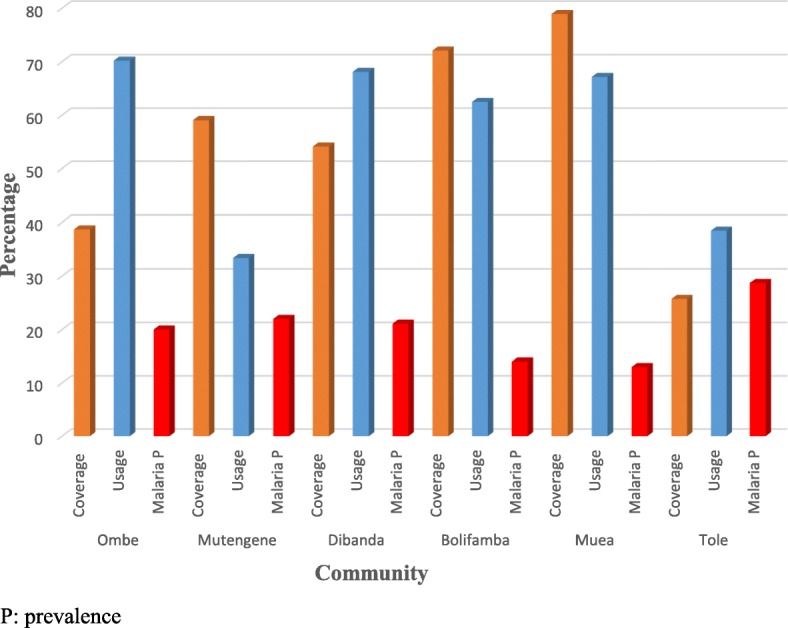
Percentage of ITNs coverage, usages and malaria prevalence across communities

The overall prevalence of malaria parasitemia in the study population was 20.1%. In a binary logistic regression, after adjusting for age group and sex it was observed that the likelihood of having malaria infection was decreased by 32.7 and 57.4% among irregularly and regular ITNs users respectively, when compared with non-users. However, the likelihood of having malaria infection was only significantly reduced among regular ITNs users (*p* = 0.036) as shown on Table 6. From Fig. 4 it was observed that Tole with the least coverage and usage of ITNs (25.7 and 38.35% respectively) had the highest prevalence of malaria parasitemia while Muea and Bolifamba with the higher coverage (78.9 and 72.1% respectively) and usage (67.2 and 62.5% respectively) of ITNs had the least prevalence of malaria parasitemia. In a binary correlation between ITN coverage and the prevalence of malaria parasitemia it was showed that the prevalence of malaria decreases with increase in ITNs coverage (*r* = − 0.899, *p* = 0.015). On the other hand, binary correlation between usage and the prevalence of malaria parasitemia also indicated that the prevalence of malaria decreases with an increase in ITN usage, though not significantly (*r* = − 0.641, *p* = 0.170) as detailed out in Table 7.

**Table 6:** Relationship between bed net usage and the prevalence of malaria parasitemia

Status of ITNs usage	Number individuals enrolled	Number positive (%)	Adjusted OR	*P* value
Non users	278	65 (23.3)	1	
Irregular users	66	13 (19.7)	0.673 (0.391–1.487)	0.426
Regular users	466	83 (17.8)	0.426 (0.467–0.972)	0.036
Overall	800	161 (20.1)		

NB *Non users is the control group (*1)

**Table 7:** Correlation matrix of ITN coverage, usage and the prevalence of malaria

		Coverage	Usage	malaria p
Coverage	Pearson Correlation	1	0.341	−0.899
	P, value	–	0.508	0.015
Usage	Pearson Correlation	0.341	1	−0.641
	P, value	0.508	–	0.170
malaria p	Pearson Correlation	−0.899	−0.641	1
	P, value	0.015	0.170	–

## Discussion

The aim of this study was to determine the coverage of ITNs, usage rate and factors associated to ITN usage among residents of malaria endemic communities within the Mount Cameroon area. The study also examined the effect of ITNs coverage and usage on the prevalence of malaria parasitemia. ITN coverage was expressed in three ways: the number of ITNs per bed space, average number of ITNs per household and the proportion of households with at least one ITN. Ombe and Tole had the least ITNs coverage. This can be explained by the fact that Ombe and Tole are rural communities which never had ITN distribution points during massive distribution campaigns. Thus, during ITNs distribution they probably did not have the same chance of receiving nets compared to people resident in the semi-urban communities. This observation was in agreement with previous studies [25–28] that demonstrated consistent inequities between urban and rural populations during bed nets distribution campaigns.

The 58.5% coverage of bed spaces was very low with respect to the Ministry of Public Health’s ambition to achieve 100% coverage of bed spaces in 2015 [29]. Nonetheless, the 77.6% coverage of households recorded was higher than the 59.7% reported earlier [3] and 69.3% [5]. The difference here may be due to the continuous effort of the national malaria control program to foster the use of ITNs.

The proportion of ownership versus regular usage (77.6% Vs 58.0%) points to the problem of noncompliance that was earlier shown by other studies in, Kenya, Tanzania Nigeria and in Yaounde, Cameroon [3, 5, 9–11].

Of the total number of ITNs found in the communities, 53.6% of the ITNs were torn, probably due to the fact that most of the nets were acquired about 3 years ago (during the nationwide distribution campaign) and have therefore been in use for long. Furthermore, most of the sensitization talks given to the population are focused more on creating awareness about the benefits of ITNs usage and very little is usually said about its maintenance. The highest number of torn nets found in Dibanda can be explained by the fact that usage was higher in Dibanda coupled to the fact that Dibanda had the least number of nets in package.

Tole had the highest number of nets in package (52.6%). This may be attributed to ignorance on the part of the population which is dominated by individuals who have attended at most primary level of education. In addition, Tole lacks a fully functional health facility which plays an important role in the sensitization of the population especially during antenatal consultations (ANC).

Amongst the reasons advanced by the participants for not using their ITNs, ‘too hot/feel heat in’ and ‘No mosquitoes were the most frequent reasons. This finding is consistent with previous reports [9, 11]. When the reasons for not using ITNs were analyzed per community, 60% of the participants in Ombe reported that ‘hot/feel heat in the net’ was the reason why they failed to sleep under the nets even during the rainy reason. A probable reason for this is the fact that Ombe is found at lowest altitude and is therefore, hotter than the other communities. In Mutengene the perception of no mosquitoes was the main reason for not using bed nets. This can be accounted for by the fact that Mutengene had the highest number of cement block houses. Occupants in cement block houses may be misled by the fact that everywhere is sealed which will prevent mosquitoes from entering the house. This finding was further supported by the relationship observed between ITN usage and nature of household.

Previous studies have shown that ITNs usage is associated with level of education, age and gender [3, 30–32]. This study found no association between ITNs usage and these factors. This contradiction may be explained partially by the fact that in the past malaria control strategies gave more attention to vulnerable groups like children below 5 years and pregnant woman. Besides, public health awareness campaign have educated people on the benefits of using ITNs as a tool of malaria prevention. Furthermore, the recent move towards universal coverage which has been strengthened through repeated massive distribution of ITNs must have been minimized these discrepancies.

It was observed that ITN usage increases with an increase in number of occupants per household. This is because net usage depends on the likelihood of the user being sensitized on its benefits. The greater the number of occupants in a household, the more the chances of having someone who can educate the others on the benefits of ITN usage.

A previous study [33] reported that the prevalence of malaria parasitemia decreases with increasing altitude. In contrast, results of this study demonstrated that Tole which is at the highest altitude had the highest prevalence of malaria parasitemia. This discrepancy can be partially explained by the fact that Tole had the lowest coverage of ITNs (only one out of every four bed spaces had a ITN and about five out of every ten households had at least bed net). This finding was in accordance with another study of [34] which demonstrated that malaria parasitemia prevalence varies with ITNs coverage.

The highest prevalence of malaria parasitemia was recorded among non-ITN users while regular ITN users had the least prevalence of malaria parasitemia. A possible explanation can be the difference in exposure of these individuals to the malaria vectors. Thus, fewer mosquitoes bites means fewer inoculations and therefore, fewer malaria infection episodes. This was in agreement with a previous report [7] which stipulated that in order to achieve the full benefits of bed nets, individuals need to own and use them properly and regularly.

## Conclusion

Despite the efforts made to scale up ITN distribution so that universal coverage can be attained, coverage remains low. ITN usage was affected by the number occupants per household, altitude and the nature of house while ‘too hot/feel heat in’ and ‘No mosquitoes’ were the most frequent reasons for not using ITNs. The prevalence of malaria parasitemia was reduced by more than 50% among regular ITNs users while an while increased in ITN coverage significantly reduces malaria prevalence. It is therefore evident that in order to achieve the full benefits of ITNs as a tool of vector control and as a strategy to cut down the prevalence of malaria parasitemia, coverage needs to be increased and a mechanism put in place to replace torn nets. The entire population should be educated on the benefits of regular usage and maintenance of bed nets.

## Additional files

Additional file 1: Questionnaire develop and used for the study (DOCX 15 kb)

Additional file 2: Raw data collected for the e study (SAV 36 kb)

## Data Availability

All the data collected that supports the findings of this study has been presented in the manuscript. However, the raw data has been made available (Additional file 2).

## Abbreviations

ACTs: Artemisinin-based combination Therapies
ANC: Antenatal Consultations
EIR: Entomological Inoculation Rates
ITNs: Insecticide-treated nets
NMCPN: National Malaria Control Program
WHO: World Health Organisation
