# Supplementary material for: Coverage and usage of insecticide treated nets (ITNs) within households: associated factors and effect on the prevalance of malaria parasitemia in the Mount Cameroon area
Source: BMC Public Health. 2019 Sep 3;19:1216. doi: 10.1186/s12889-019-7555-x (PMC6724238; doi:10.1186/s12889-019-7555-x)
Supplement: Supplementary file 1 — Questionnaire develop and used for the study (DOCX 15 kb) [file 12889_2019_7555_MOESM1_ESM.docx]

APPENDIX

**QUESTIONNAIRE.**

| Site Name | Household number | Number of occupants in the household | Nature of the house (1- cement block, 2- wooden house) | Page number |
| --- | --- | --- | --- | --- |
|  |  |  |  |  |

1. Name of the family head……………………………………………………………………
2. Date of enrolment………………………/…………………………/………………………
3. Inform consent obtained………………………………………………………… (Yes/No)

**Section 1**: **Information about the household (head of household or adult > 18 years)**

1. What is the highest level of education of the head of the household?....1-.Primary school 2-Secondary school 3-Higher education 4-Other, specify
2. How many sleeping places do you have in your house hold……………….
3. Do your household have bed nets…………………………………………… (Yes/No)
4. If yes how many I____I
5. If yes for 6 where did you get your bed net (1-ANC 2-Bought 3-massive distribution 4-

5-Oher, specify.................

1. When was the net was obtained…../ 1-<1month, 2- <3month, 3-<6month, 4- <1 yrs 5->1yrs

**Section 2: Status of the bed net.**

1. Have you impregnated the bed net since you last obtained it ………………. (Yes/No)
2. If yes when was this………. 1-<1month, 2- <3month, 3-<6month, 4- <1 yrs 5->1yrs
3. What is your nets used for……1- use to sleep under, 2-still in package, 3-used for other purpose, specify ……………………….
4. If 12 is (2), can it be used for sleeping under...................................................... ( Yes/No)
5. If No, why this is net no longer available for sleeping under in the household……

1-Net was damaged and thrown away 2-Net was given away to others 3-Net was stolen 4-Net was sold

1. How many months ago did this net become unavailable for sleeping under in the household? …….1-.0–6 months, 2-.> 6 months, 3-Don’t know
2. What is the nature of your bed net under used………………………...…..1- good, 2-Torn

**Section 3: Bed net use and handling**

1. When did you start using net…..? (1- Before the massive distribution of bed nets in 2011, 2-After the distribution of bed nets in 2011)
2. Who are those using the net…………………… 1-<5yrs, 2-5 to15yrs, 3->15yrs 4-sex…..M……….F, 5-everybody in the household.
3. Was this net used last night to sleep under…................................................ (Yes/No)
4. If No, why was this net not used last night……….1-Too hot 2-Don’t like the smell 3-Feel “closed in” 4-No malaria now 5-No mosquitoes 5-The net is too torn or old 6-Used another net 7-User did not sleep her 8- Don’t know
5. During which periods of the year is this net used to sleep under……. 1... All year, 2....Only the rainy seasons, 3....Only the dry season, 4 ....Don’t know
6. Do you tuck the net in at night...............................................................................(Yes/No)

**MORBIDITY MONITORING QUESTIONNAIRE**

| Site Name | House hold number | Number of individuals in the household | Date of visit | Participant status | |
| --- | --- | --- | --- | --- | --- |
|  |  |  |  | Number using nets | Number not using nets |
|  |  |  |  |  |  |

1. In the past week, how often did you used the bed net……. 1-Every night (7 nights),2-Most nights (5–6 nights) 3-Some nights (1–4) 4-Not used at all (0 nights) 5-Don’t know
2. Did all those using bed net slept under the net last night…………..…………..( yes/No)
3. If No who are those who didn’t ……..1-children,-<5yrs 2-children 5-15yrs 3-adult >15yrs
4. If No for 2 why did they not use the net………….1-Too hot 2-No malaria now 3-No mosquitoes now 4- Don’t know
5. Has any person had fever after our last visit………………….…………………(Yes/NO)
6. If yes did the person report to the Hospital ……………………………………….(Yes /No)
7. If yes for 6 check his/her hospital data to see the cause of fever…………(1=malaria 2=others)
8. If auto medication taken, what medication was taken…….…(1=Anti malaria,2= others)
